# The association between upper tarsal conjunctiva appearance and corneal biomechanical weakening in refractive surgery candidates

**DOI:** 10.1038/s41598-025-00122-2

**Published:** 2025-04-28

**Authors:** Phit Upaphong, Napaporn Tananuvat, Muanploy Niparugs, Janejit Choovuthayakorn, Chulaluck Tangmonkongvoragul, Somsanguan Ausayakhun, Winai Chaidaroon

**Affiliations:** 1 Department of Ophthalmology, Faculty of Medicine, Chiang Mai University, Chiang Mai, Thailand; 2 Chiang Mai University LASIK Center, Center of Medical Excellence, Faculty of Medicine, Chiang Mai University, Chiang Mai, Thailand

**Keywords:** Allergic conjunctivitis, Conjunctival roughness, Corneal deformation dynamics, Ectasia, Ectatic corneal disease, Papillae, Eye diseases, Predictive markers

## Abstract

Current evidence suggests allergic conjunctivitis and contact lens use may affect corneal biomechanics, but the relationship between conjunctival changes and biomechanical abnormalities remains unclear. This study aimed to determine whether upper tarsal conjunctival roughness and redness are associated with corneal biomechanical weakening in refractive surgery candidates without ectatic corneal disease. Conducted at Chiang Mai University LASIK Center between July 2023 and January 2024, conjunctival roughness and redness were graded using the Institute for Eye Research (IER) scales, while corneal biomechanics were assessed through Corvis parameters. The study included 434 eyes from 217 participants (70.5% female), with a mean age of 33.4 years (SD 8.3). Ocular allergy was reported by 23.0% (50/217) of participants, and 54.4% (236/434) had a history of contact lens use. The median conjunctival roughness score was 2 (IQR 0, 5). Conjunctival roughness score was positively correlated with biomechanical indices, particularly the deformation amplitude (DA) ratio (coefficient 0.009, 95% confidence interval 0.001 to 0.017, *p* = .04), suggesting weakened biomechanical property. No significant association was found between conjunctival redness and corneal biomechanics. In conclusion, upper tarsal roughness may associate with biomechanical weakening of the cornea, though these changes were insufficient to significantly affect the Corvis Biomechanical Index (CBI).

**Supplementary Information:**

The online version contains supplementary material available at 10.1038/s41598-025-00122-2.

## Introduction

Corneal biomechanics studies the cornea’s response to forces, focusing on the deformation of corneal tissue and predicting its behavior under various conditions. This provides insights into the cornea’s dynamic responses in both physiological and pathological states^1^. Corvis Scheimpflug Technology (Corvis ST) measures corneal deformation in real-time, offering several new deformation-dynamic corneal response (DCR) parameters beyond those from the Ocular Response Analyzer (ORA)^2,3^.

Ectatic corneal disease (ECD) such as keratoconus is characterized by the progressive loss of collagen fibril orientation and biomechanical weakening in the corneal stroma^4^, but its etiology remains unclear^4^. Corneal laser refractive surgeries, such as laser-assisted in situ keratomileusis (LASIK) and photorefractive keratectomy (PRK), are designed to correct refractive errors but can reduce the biomechanical strength of the cornea^5^. Therefore, these procedures are contraindicated in patients with ECD^6^. Biomechanical parameters are gaining interest in the screening of refractive surgery candidates, as they help identify early corneal weakening before any external changes become apparent^2,7^. Additionally, these parameters provided added values in detecting ECD, compared with using corneal tomography/topography parameters alone^8^.

Studies identified a good correlation between Corvis biomechanical parameters and tomographic measurements in keratoconus^2,3^. Nonetheless, the lack of standardized cut-off values, due to insufficient comprehensive research defining definitive reference ranges for these biomechanical parameters and their variability across different populations, limits their use in clinical practice^9^. Additionally, subtle abnormalities in biomechanical parameters were occasionally detected despite normal corneal tomography/topography, without a clear explanation. Several conditions, such as allergic conjunctivitis, contact lens use, and floppy eyelid syndrome (FES), could affect corneal biomechanical properties^1,10–15^. It was suggested that mechanical microtrauma, irritation, and inflammatory responses lead to structural changes in the cornea, thereby compromising its strength^16,17^. However, the pathophysiology is not well understood. These conditions have common findings, including a papillary reaction of the upper tarsal conjunctiva and a resulting conjunctival irregularity. Mechanical interactions between the conjunctiva and cornea during blinking may trigger localized biomechanical modifications through direct friction or inflammatory reactions. Hence, this study was conducted to explore the association between the appearance of the upper tarsal conjunctiva and corneal biomechanical properties.

## Methods

This cross-sectional study was primarily conducted to find the association between tarsal conjunctival roughness and corneal biomechanical parameters in refractive surgery candidates whose eyes do not have ECD. The secondary objective was to explore the appearance of the tarsal conjunctiva in these cases. The study prospectively recruited participants who presented for screening at the Chiang Mai University LASIK Center between July 2023 and January 2024. Both eyes of the participants were included. The study protocol was approved by the Research Ethics Committee, Faculty of Medicine, Chiang Mai University (study code: OPT-2566-09505), and adhered to the principles of the Declaration of Helsinki. Written informed consent was obtained from all participants.

Participants were eligible if they were aged 18 years or older, with or without contact lens use. Exclusion criteria included the presence of significant corneal scars, pterygium, corneal surface irregularities, inflammatory or infectious diseases of the cornea and sclera, collagen vascular diseases or connective tissue disorders, glaucoma or ocular hypertension, tight lids that prevented upper eyelid eversion, previous corneal or intraocular surgeries, and any eyelid surgery. Because of the known association between allergic conjunctivitis and keratoconus^7^, we excluded cases with ECD to assess whether the biomechanical change was due to the conjunctiva itself rather than the disease-induced biomechanical weaknesses.

### Clinical assessment

**Fig. 1:**
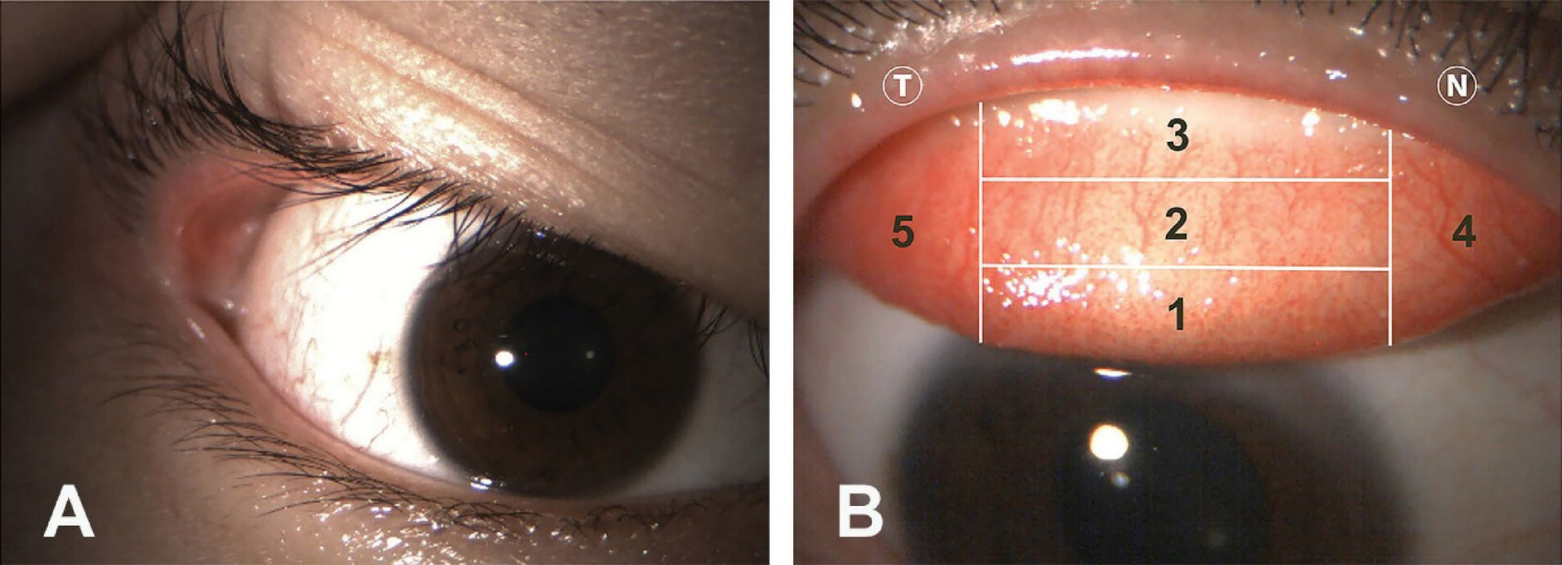
The clinical examination of the right eye revealed grade 1 floppy eyelid (**A**) and grade 1 roughness, particularly in zone 1 (**B**), with the five-zone grading system being used to evaluate the upper tarsal conjunctiva (**B**). N, nasal; T,  temporal.

The severity of floppy eyelids was graded by pulling the upper lid with the thumb and observing the visibility of the tarsal conjunctiva: grade 0 (normal) has no visibility, grade 1 (mild) showed less than one-third visibility, grade 2 (moderate) showed one-third to one-half visibility, and grade 3 (severe) showed more than half visibility^18^. As illustrated in Fig. 1-A, the patient exhibites grade 1 floppy eyelid.

The upper tarsal conjunctiva was evaluated after everting the upper eyelid. The appearances were graded using the Institute for Eye Research (IER) grading scales under standardized conditions with diffuse yellow illumination at 100% intensity through a diffusion filter without fluorescein staining^19^. The upper tarsal conjunctiva was divided into five zones (Fig. 1B). Each zone was assessed on a five-point scale (0–4) for roughness and redness/vascularization^19^, which were summed to create total roughness and redness scores. Additionally, the presence of scars in each zone was also recorded. Figure 1B demonstrates grade 1 roughness, particularly in zone 1.

### Corneal imaging techniques

Corneal biomechanics were analyzed using the Corneal Visualization Scheimpflug Technology (Corvis ST, Oculus Optikgeräte GmbH, Germany), software version 1.6r2031. The recorded biomechanical parameters included: (1) the velocity of the corneal apex at the first inward applanation (A1 velocity); (2) the maximal ratio of the central deformation to the average deformation 2 mm on either side of the center (DA ratio), which compares the amount of deformation at the center to that of nearby points when pressure is applied; (3) the integrated sum of the reciprocal of the radius between the inward and outward applanation (integrated radius, IR), which measures corneal stiffness, with a larger area under the curve indicating a softer cornea; (4) the resultant pressure at the first inward applanation divided by corneal displacement compared to the undeformed cornea (SP-A1), which quantifies how the cornea responds to pressure when it first flattens; and (5) the thinnest corneal point relative to pachymetric progression indices (ARTh). Additional parameters, such as the Stress-Strain Index (SSI), which quantifies how much the cornea deforms (strain) under a given amount of force or stress; Corvis Biomechanical Index (CBI); Tomographic and Biomechanical Index (TBI); as well as biomechanically corrected intraocular pressure (bIOP), were also obtained from the Corvis ST. Corneal topography and tomography were analyzed using Pentacam (Oculus Optikgeräte GmbH, Germany) software version 1.28r06. Parameters obtained from the Pentacam included: (1) keratometry (K); (2) central corneal thickness (CCT); (3) Belin/Ambrósio enhanced ectasia total deviation index (BADD); (4) keratoconus percentage index (KISA%), a composite index used to assess the risk of keratoconus, calculated by multiplying 1/3 by K, the interior-superior index (I-S), corneal simulated astigmatism (AST), and skewed radial axes (SRAX); (5) index of surface variance (ISV), which measures the irregularity of the corneal surface; (6) index of vertical asymmetry (IVA), which evaluates the difference in steepness between the superior and inferior halves of the cornea; and (7) index of height decentration (IHD), which measures how off-center the corneal elevation is compared to the ideal centered dome shape. Only high-quality examinations that passed the software quality checks were selected, and the median values of the parameters from the three that passed quality checking were used for analysis.

### Statistical analysis

Descriptive statistics was used to report continuous variables as mean and standard deviation (SD) or median and interquartile range (IQR), depending on the distribution of the data. To address the correlation between the two eyes, a mixed-effects model analysis adjusted for bIOP, CCT, KISA%, ISV, IVA and IHD was used as a multivariable regression method. This approach aimed to identify associations between upper tarsal conjunctiva parameters and demographics, as well as biomechanical variables. Statistical significance was defined as a P-value less than 0.05.

## Results

A total of 434 eyes from 217 participants screened for refractive surgery between July 2023 and January 2024 were included. Participant demographics and ocular parameters are detailed in Table 1. The majority of participants were female (153/217, 70.5%). The mean age (SD) was 33.4 (8.3) years. About one-fourth of the participants (50/217, 23.0%) reported a history of ocular allergy. Contact lens use was observed in approximately half of the eyes (236/434, 54.4%), with a median (interquartile range; IQR) duration of 2 (0,10) years.

**Table 1:** Participant demographics and ocular parameters.

Participant demographics	(*N* = 217 cases)
Age; years, mean (SD)	33.40 (8.28)
Female; n (%)	306 (70.5)
Ocular allergy; n (%)	50 (23.0)

Ocular parameters	(*N* = 434 eyes)
Contact lens use; n (%)	236 (55.4)
Spherical equivalence; D, median (IQR)	-4.38 (-6.13, -2.75)
Maximal keratometry; D, mean (SD)	44.59 (1.48)
Minimal keratometry; D, mean (SD)	43.06 (1.42)
Average keratometry; D, mean (SD)	43.8 (1.36)
Central corneal thickness; micron, mean (SD)	530.02 (31.12)
bIOP; mmHg, mean (SD)	14.96 (1.86)
KISA%; median (IQR)	3.6 (1.60, 7.07)
ISV; mean (SD)	19.43 (7.13)
IVA; median (IQR)	0.12 (0.09, 0.15)
IHD; median (IQR)	0.01 (0.01, 0.01)
BADD; median (IQR)	1.18 (0.74, 1.57)
CBI; median (IQR)	0.09 (0.04, 0.23)
TBI; median (IQR)	0.23 (0.04, 0.46)
SSI; mean (SD)	0.97 (0.15)
DA ratio max at 2 mm; mean (SD)	4 (3.7, 4.2)
Integrated radius; mean (SD)	8.05 (1.03)
ARTh; median (IQR)	578.15 (504.60, 654.20)
SPA1; mean (SD)	107.15 (18.39)

ARTh, Ambrósio’s relational thickness; BADD,  Belin/Ambrosio enhanced ectasia display; bIOP,  biomechanically corrected intraocular pressure; CBI,  Corvis biomechanical index; D,  diopters; DA,  deformation amplitude ratio; IHD, index of height decentration; IQR,  interquartile range; IR,  integrated radius; ISV, index of surface variance; IVA, index of vertical asymmetry; KISA%, keratoconus percentage index; SD, standard deviation; SP-A1, stiffness parameter at first applanation; SSI, stress-strain index; TBI, tomographic and biomechanical index.

The upper tarsal conjunctiva showed interesting characteristics across different zones (Table 2). Overall, the median (IQR) of the total conjunctival roughness score was 2 (0, 5), with the highest roughness observed in zones 1, 4, and 5. The median (IQR) of the total conjunctival redness score was 5 (4, 8), distributed equally across all zones. Notably, scarring presented in 55 eyes (12.7%), with zone 1 displaying the most significant scarring. FES was detected in 126/434 (29.0%) eyes, with 116/434 (26.7%) having grade 1 floppy eyelid and 10/434 (2.3%) having grade 2 floppy eyelid. Table 3 presents a breakdown of upper tarsal conjunctival characteristics across four groups: contact lens users, individuals with ocular allergies, those diagnosed with FES, and a group without these conditions. The data captures conjunctival roughness, significant redness (defined as 6 or above), and the extent of scarring within each category.

**Table 2:** Clinical findings of the upper tarsal conjunctiva.

Clinical findings (*N* = 434 eyes)	Total*	Zone 1	Zone 2	Zone 3	Zone 4	Zone 5
Roughness score; median (IQR)	2 (0, 5)	1 (0, 2)	0 (0, 0)	0 (0, 0)	1 (0, 1)	1 (0, 2)
Redness score; median (IQR)	5 (4, 8)	1 (1, 2)	1 (1, 2)	1 (1, 2)	1 (1, 2)	1 (1, 2)
Scarring; n (%)	55 (12.7)	50 (11.5)	8 (1.8)	4 (0.9)	11 (2.5)	7 (1.6)

IQR, interquartile range.

*For roughness and redness scores, the total represents the combined score across all zones, while for scarring, it indicates the number of eyes with scarring in at least one zone.

**Table 3:** Upper tarsal conjunctival findings categorized by contact lens use, ocular allergy, floppy eyelid syndrome, and those without any of these three conditions.

Clinical findings	Contact lens use (*N* = 236 eyes)	Ocular allergy (*N* = 100 eyes)	Floppy eyelid (*N* = 126 eyes)	None of these three conditions (*N* = 96 eyes)
Presence of conjunctival roughness; n (%)	168	75	103	56
	(71.2)	(75.0)	(81.7)	(58.3)
Total redness score > 5; n (%)	113	54	72	48
	(47.9)	(54.0)	(57.0)	(50.0)
Presence of conjunctival scarring; n (%)	34	16	20	7
	(14.4)	(16.0)	(15.9)	(7.3)

The multivariable analysis of the biomechanical indices (Table 4) revealed that CBI was associated with age (coefficient − 0.002, 95% CI 0.003 to − 0.0, *p* = .03) and contact lens use (coefficient 0.048, 95% CI 0.021 to 0.075, *p* = .001). SP-A1 was associated with age (coefficient − 0.163, 95% CI 0.027 to 0.298, *p* = .02). IR was associated with contact lens use (coefficient 0.173, 95% CI 0.034 to 0.312, *p* = .01) and floppy eyelid (coefficient 0.182, 95% CI 0.026 to 0.337, *p* = .02). DA ratio was associated with total conjunctival roughness score (coefficient 0.009, 95% CI 0.001 to 0.017, *p* = .04). Finally, SSI was associated with age (coefficient 0.004, 95% CI 0.002 to 0.005, *p* < .001), contact lens use (coefficient − 0.049, 95% CI − 0.076 to -0.023, *p* < .001) and floppy eyelid (coefficient − 0.041, 95% CI − 0.071 to − 0.012, *p* = .006).

**Table 4:** Multivariable analysis showing the association between the Biomechanical indices and demographics, as well as ocular parameters.

Parameters	CBI		SP-A1		ARTh		IR		DA ratio (2 mm)		SSI	
	Coef.	95% CI	Coef.	95% CI	Coef.	95% CI	Coef.	95% CI	Coef.	95% CI	Coef.	95% CI
Age	− 0.002	− 0.003 to − 0.0	0.163	0.027 to 0.298	− 0.008	− 3.093 to 3.077	− 0.007	− 0.015 to 0.002	0	− 0.003 to 0.003	0.004	0.002 to 0.005
	*p* = .03		*p* = .02		*p* = 1.00		*p* = .12		*p* = .87		*p* < .001	
Female	− 0.001	− 0.031 to 0.029	− 0.794	− 3.278 to 1.69	1.921	− 54.639 to 58.48	− 0.046	− 0.202 to 0.11	− 0.011	− 0.068 to 0.046	0.012	− 0.018 to 0.041
	*p* = .93		*p* = .53		*p* = .95		*p* = .56		*p* = .87		*p* = .44	
Ocular allergy	− 0.001	− 0.031 to 0.03	− 1.097	− 3.641 to 1.447	− 17.349	− 75.266 to 40.568	0.048	− 0.111 to 0.208	− 0.016	− 0.074 to 0.042	0.022	− 0.008 to 0.053
	*p* = .97		*p* = .40		*p* = .56		*p* = .55		*p* = .59		*p* = .15	
Contact lens use	0.048	0.021 to 0.075	− 0.127	− 2.35 to 2.096	20.277	− 30.338 to 70.893	0.173	0.034 to 0.312	0.051	0.001 to 0.102	− 0.049	− 0.076 to − 0.023
	*p* = .001		*p* = .91		*p* = .43		*p* = .01		*p* = .05		*p* < .001	
Floppy eyelid	0.005	− 0.026 to 0.035	− 1.173	− 3.657 to 1.312	− 6.981	− 63.55 to 49.588	0.182	0.026 to 0.337	0.057	0.001 to 0.114	− 0.041	− 0.071 to − 0.012
	*p* = .77		*p* = .35		*p* = .81		*p* = .02		*p* = .05		*p* = .006	
Total conjunctival roughness score	0	− 0.004 to 0.005	− 0.029	− 0.393 to 0.336	0.12	− 8.169 to 8.409	− 0.009	− 0.031 to 0.014	0.009	0.001 to 0.017	− 0.001	− 0.005 to 0.004
	*p* = .91		*p* = .88		*p* = .98		*p* = .46		*p* = .04		*p* = .79	
Total conjunctival redness score	0.004	− 0.001 to 0.008	− 0.1	− 0.462 to 0.262	− 0.115	− 8.365 to 8.135	0.005	− 0.017 to 0.028	0.001	− 0.007 to 0.01	0.001	− 0.004 to 0.004
	*p* = .11		*p* = .59		*p* = .98		*p* = .64		*p* = .73		*p* = .98	

ARTh,  Ambrósio’s relational thickness; CBI,  Corvis biomechanical index; CI,  confidence interval; Coef., coefficient; DA,  deformation amplitude; IR,  integrated radius; SP-A1,  stiffness parameter at first applanation; SSI,  stress-strain index.

## Discussion

**Fig. 2:**
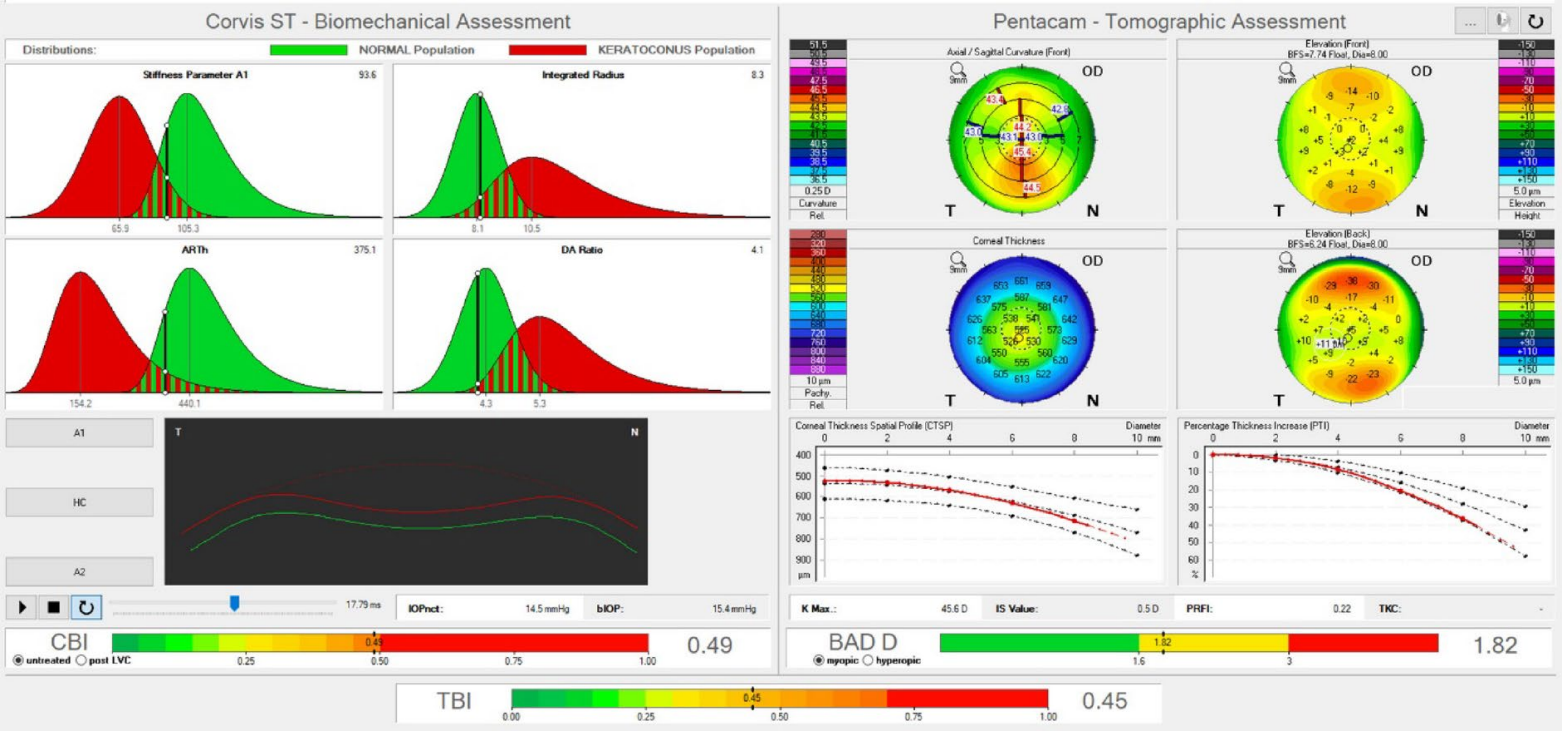
The Corvis-ST Tomographic Biomechanical Ambrósio, Roberts, Vinciguerra (ARV) Display shows borderline changes in the Corvis Biomechanical Index (CBI), the Belin/Ambrósio enhanced ectasia total deviation index (BADD), and the Tomographic and Biomechanical Index (TBI). Image generated using the Pentacam (Oculus Optikgeräte GmbH, Germany, software version 1.28r06) and the Corneal Visualization Scheimpflug Technology (Corvis ST, Oculus Optikgeräte GmbH, Germany, software version 1.6r2031, www.oculus.de).

Screening for ECD before performing refractive surgery is crucial, as comprehensive ocular examinations and imaging are necessary to exclude ECD. Interestingly, our previous study found that up to 45.72% did not pass the screening due to abnormal corneal topography or corneal tomography detected from corneal imaging^20^. Specifically, clinical keratoconus was identified in 1.65%, and keratoconus suspect was identified in 28.88% of cases^20^. Advances in technology for assessing corneal biomechanics help in the early detection of ECD but also lead to an increase in borderline cases. Even when the gross appearance and standard ophthalmic indices seemed normal, subtle abnormalities in biomechanical parameters such as the CBI and TBI might be detected without a clear explanation. For instance, a 20-year-old myopic female had not used contact lenses and reported no history of allergy or symptoms associated with ocular allergy. Nevertheless, clinical examination revealed grade 1 floppy eyelid (Fig. 1A) and grade 1 superior tarsal roughness, especially in zone 1 (Fig. 1B) and. Interestingly, screening revealed an increase in CBI and TBI in the right eye (Fig. 2), without significant abnormalities from corneal tomography/topography (Supplementary Figs. 1 and 2). Only mild increased BADD was detected. Other standard indices were normal, except for the pachymetric progression (Dp) (Supplementary Figs. 2 and 3). The left eye also showed similar clinical and imaging findings.

Our results suggested that conjunctival roughness due to upper tarsal papillae may associated with the subtle biomechanical changes, specifically an increase in DA ratio at 2 mm., indicating softer tissue^1^. This finding supported that biomechanical weakness could be detected before changes in corneal tomography and topography in patients prone to ECD, as demonstrated by the study case. Nonetheless, these conjunctival changes were not substantial enough to alter the CBI, which integrated various biomechanical parameters, and the TBI, which integrated data from corneal tomography and corneal biomechanics.

Conditions such as allergic conjunctivitis, contact lens use, and FES were known to cause papillary reactions in the upper tarsal conjunctiva ​and could lead to biomechanical weakness of the cornea^1,10–15^. The prevalence of ECD, including keratoconus, increased in patients with allergic conjunctivitis^21^. In a study, patients with allergic conjunctivitis showed significantly higher indices of corneal ectatic changes, including the index of surface variance (ISV), index of vertical asymmetry (IVA), keratoconus index (KI), index of height decentration (IHD), and BADD^12^. Furthermore, patients with both keratoconus and atopy had steeper and thinner corneas than their counterparts with keratoconus but no atopy^16^. Consequently, allergic conjunctivitis was identified as a factor associated with the progression of keratoconus^22^. Our findings showed that age associated with CBI, SP-A1 and SSI. The correlation between younger age and increased CBI as well as decreased SSI might support the conclusions of a meta-analysis indicating a higher risk of keratoconus progression in younger individuals^23^.

The connection between allergic conjunctivitis and ECD, or its progression, was hypothesized to be due to eye rubbing in response to the itching and discomfort from allergic reactions^16^. Eye rubbing damaged the corneal epithelium, which triggered the release of matrix metalloproteinase and inflammatory cytokines, causing protein decomposition in the cornea and leading to corneal deformation^16^. This hypothesis was supported by findings that indicated a relationship between the dominant hand and the eye with more advanced keratoconus^16^. Although these changes were not well understood, biomechanical parameters might be used to detect early internal changes such as the elasticity and deformation of the cornea before noticeable external changes, such as curvature and thickness changes^7^. However, information on whether conjunctival roughness causes long-term weakening of the cornea was limited.

Notably, in chronic ocular allergy, conjunctival papillae might persist even in the absence of symptoms and typical inflammatory signs. However, this study did not find an association between ocular allergy and biomechanical changes. In addition to the weakening of corneal strength by cytokines released during inflammatory and allergic reactions, we hypothesized that the friction caused by the roughness of the upper tarsal conjunctiva moving across the cornea during each blink might contribute to subtle changes in corneal biomechanics, either mechanically or through inflammatory responses.

Contact lenses were another factor that induces changes in the tarsal conjunctiva, such as increased redness and roughness, possibly due to allergenic stimuli, contact lens solutions and mechanical irritation^10^. Our study found that contact lens use was associated with increases in CBI and IR, along with a decrease in SSI, indicating compromised corneal biomechanics. Likewise, previous studies showed that contact lenses might alter the corneal biomechanical properties such as corneal hysteresis (CH), corneal resistance factor (CRF) and DA ratio^1,13,14^. The mechanism behind the weaker tissue was that soft contact lenses might cause stromal corneal edema, which widened the space between collagen fibrils^24^. Additionally, contact lens could induce local alternation of inflammatory cytokines and chemokines^17^. Consequently, chronic contact lens usage triggered corneal remodeling and changes in corneal biomechanics^17^.

Similarly, eyelid problems such as FES might associate with compromised corneal strength as indicated by a decrease in CH in a study^15^. Our findings revealed that FES correlated with an increase in IR, and a decrease in SSI, reflecting compromised corneal biomechanics. The probable mechanism involved the flaccid eyelids being easily everted during sleep, causing mechanical contact with bed sheets and subsequent corneal and conjunctival irritation^25^. This effect could lead to corneal collagen crosslinking failure in eyes with keratoconus, suggesting that the failure was probably due to a biomechanical effect rather than a systemic alteration^25^.

The predominance of female participants, the younger age group, and the focus on individuals with refractive errors might limit the generalizability of our findings. However, we included age and sex as variables in the multivariable regression equation.

In conclusion, this study highlighted the possible impact of upper tarsal roughness on the weakening of corneal biomechanics, which were notably prevalent among individuals using contact lenses or with ocular allergy. The most affected biomechanical parameters were DA ratio at 2 mm. However, these changes were insufficient to alter the TBI and CBI. This finding might partially explain the link between chronic allergic conjunctivitis and keratoconus, and clarify subtle corneal biomechanical abnormalities despite normal results from corneal tomography or topography. Nevertheless, future cohort studies are necessary to explore the long-term impact of conjunctival changes on corneal biomechanics.

## Electronic supplementary material

Below is the link to the electronic supplementary material.

Supplementary Material 1

## Data Availability

The datasets used and analyzed during the current study are available from the corresponding author upon reasonable request.
