# Supplementary material for: The association between upper tarsal conjunctiva appearance and corneal biomechanical weakening in refractive surgery candidates
Source: Sci Rep. 2025 Apr 28;15:14793. doi: 10.1038/s41598-025-00122-2 (PMC12037714; doi:10.1038/s41598-025-00122-2)
Supplement: Supplementary file 1 — Supplementary Material 1 [file 41598_2025_122_MOESM1_ESM.docx]

Supplementary Material

## Supplementary Figures


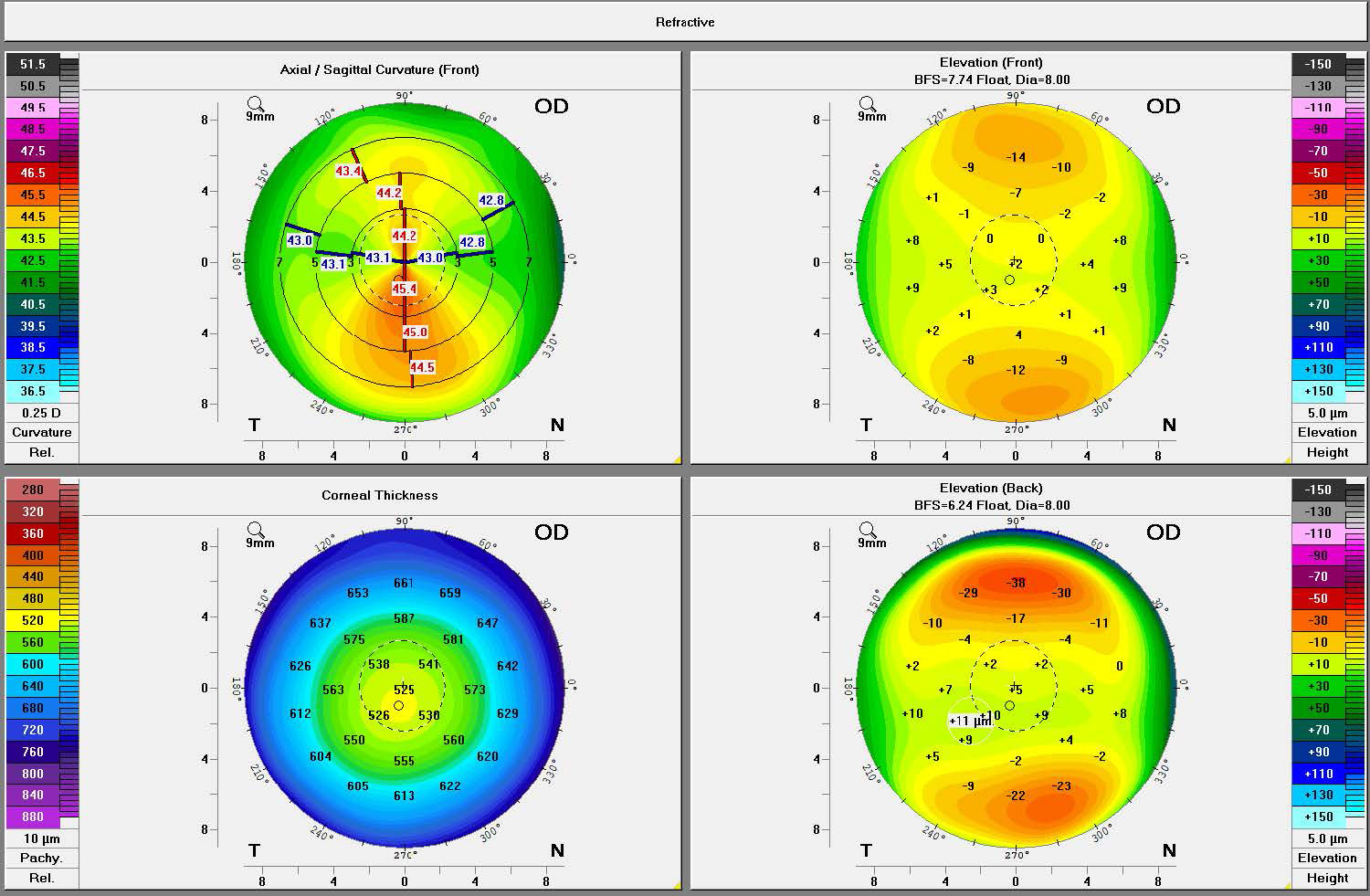


**Supplementary Figure 1** 4-composite refractive map of Pentacam. The image was created using the Pentacam (Oculus Optikgeräte GmbH, Germany, software version 1.28r06; www.oculus.de).


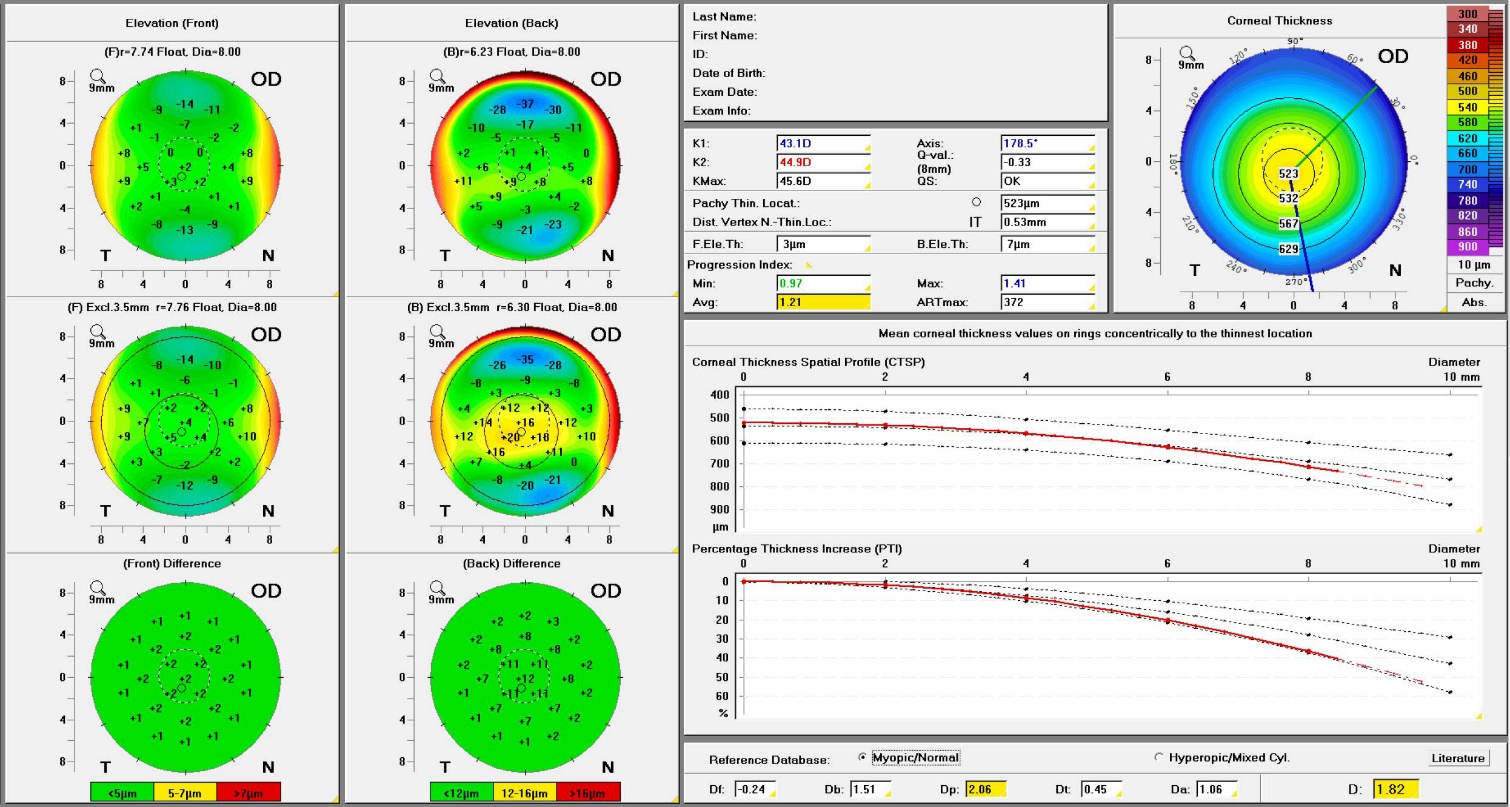


**Supplementary Figure 2** Belin/ Ambrósio enhanced ectasia display of Pentacam. The image was created using the Pentacam (Oculus Optikgeräte GmbH, Germany, software version 1.28r06; www.oculus.de).


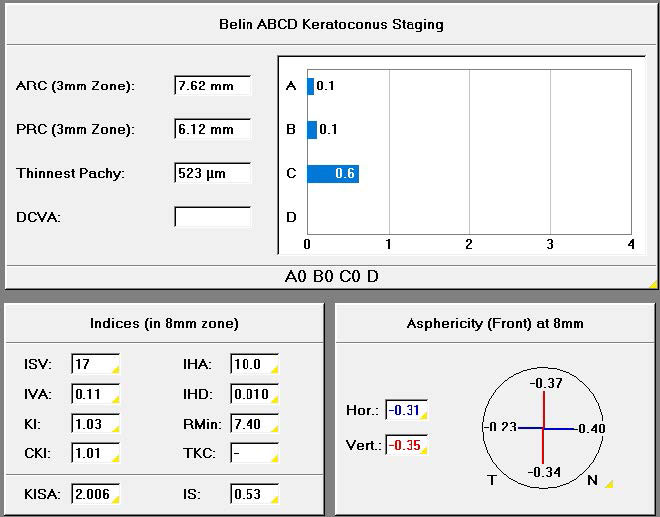


**Supplementary Figure 3** Topometric indices and Topographical Keratoconus Classification (TKC) from topometric/ TKC display of Pentacam. The image was created using the Pentacam (Oculus Optikgeräte GmbH, Germany, software version 1.28r06; www.oculus.de).
